# Industrial Coagglomeration, Green Innovation, and Manufacturing Carbon Emissions: Coagglomeration’s Dynamic Evolution Perspective

**DOI:** 10.3390/ijerph192113989

**Published:** 2022-10-27

**Authors:** Lu Zhang, Renyan Mu, Nigatu Mengesha Fentaw, Yuanfang Zhan, Feng Zhang, Jixin Zhang

**Affiliations:** 1 School of Management, Wuhan University of Technology, Wuhan 430070, China; 2 Graduate School of Engineering, Tohoku University, Sendai 980-8579, Japan; 3 Hubei Product Innovation Management Research Center, Wuhan 430070, China; 4 School of Economics and Business Administration, Central China Normal University, Wuhan 430079, China; 5 School of Economics and Management, Hubei University of Technology, Wuhan 430068, China

**Keywords:** industrial coagglomeration, green innovation, manufacturing carbon emissions, threshold effect, mediating effect, dynamic evolution

## Abstract

The achievement of China’s low-carbon development and carbon neutrality depends heavily on the decrease of manufacturing carbon emissions. From coagglomeration’s dynamic evolution perspective, by using panel-threshold-STIRPAT and mediation-STIRPAT models, this study examines the relationships among industrial coagglomeration, green innovation, and manufacturing carbon emissions and explores the direct and indirect function mechanisms. Panel data of China’s 30 provinces from 2010 to 2019 are employed. The results imply that, first, the impact of industrial coagglomeration on manufacturing carbon emissions is nonlinear and has significant threshold effects. Industrial coagglomeration negatively affects manufacturing carbon emissions, and as the coagglomeration level deepens, the negative effect has a diminishing trend in marginal utility. Once the coagglomeration degree exceeds a certain threshold, the negative impact becomes insignificant. At present, for 90% of China’s regions, an increase in industrial coagglomeration level can help reduce manufacturing carbon emissions. Second, green innovation is a vital intermediary between industrial coagglomeration and manufacturing carbon emissions. It is a partial intermediary when industrial coagglomeration is at a relatively lower-level stage and a complete intermediary when industrial coagglomeration is at a relatively higher-level stage. These findings reveal the significance of optimizing industrial coagglomeration and the level and efficiency of green innovation to decrease carbon emissions.

## 1. Introduction

Due to the increasingly serious issue of global warming, how to cut carbon emissions and eventually reach the goal of carbon neutrality has become a major concern for countries [1]. As one of the largest carbon emitters, China has placed great emphasis on decreasing carbon emissions and has formulated a series of industrial low-carbon growth plans [2]. In September 2020, it solemnly pledged to the world that it would work to reach a peak in carbon emissions by 2030 and realize carbon neutrality by 2060, namely, “dual-carbon” goals [3]. Since China is still a developing country in the process of industrialization, it needs to consider economic development while making and implementing carbon emission reduction strategies. Meanwhile, as the manufacturing industry is the main industrial source of carbon emissions [4], as well as China’s pillar of economic development, to effectively realize the “dual-carbon” and economic growth goals in a coordinated way, the low-carbon development of the manufacturing industry should thus be the top priority over all other industries. Based on this, the exploration of the manufacturing carbon emission abatement effect and its driving forces have received great concern from all walks of life [5,6,7].

Industrial coagglomeration, a new industrial agglomeration mode, is a crucial tool for China to better achieve economic green transformation by building a modern industrial system [8,9]. Its concept was initially proposed by Ellison and Glaeser in 1997 [10], which defines the spatial agglomeration phenomenon of heterogeneous industries attracting each other and co-locating. Different from single industrial agglomeration, it is not only relevant to the spatial layout of industries but also to their interior links [11,12], including the horizontal correlation formed by the sharing of the labor force and knowledge spillover between various industries, and the vertical correlation between downstream and upstream industries having an input–output relationship [12]. Taking the coagglomeration of manufacturing and producer services as an example, productive services are highly correlated with manufacturing, and their development is often dependent on that of the manufacturing sectors. The location-locking function of manufacturing attracts productive services to have economic ties with them, thus forming a contiguous spatial layout in terms of geographical location [13]. In the past decade, the Chinese government has released many measures encouraging industrial coagglomeration, particularly the combined development of manufacturing and producer services [13,14]. Effective industrial coagglomeration has been demonstrated to significantly support regional economic growth [14]. However, is it also conducive to reducing manufacturing carbon emissions? This is a crucial problem that must be examined.

The majority of the pertinent studies currently available have concentrated on how single industrial agglomeration affects carbon emissions, and rare research has involved the link between industrial coagglomeration and carbon emissions. Lu et al. [15], Xu et al. [16], Huang et al. [17], and Chen et al. [18] indicate that industrial agglomeration results in more carbon emissions. By contrast, Peng et al. [19] argue that industrial agglomeration alleviates carbon emissions through technology and knowledge spillovers, economies of scale, and industrial structure optimization. Moreover, Sun and Liu [20] point out that how industrial coagglomeration impacts carbon emission efficiency has industry heterogeneity. Meng and Xu [9] and Li et al. [21] found that industrial coagglomeration has a nonlinear influence on carbon emissions, which changes with the coagglomeration level and improper resource allocation degree. Overall, the above research primarily concentrates on how industrial agglomeration or coagglomeration affects the carbon emissions of the whole industry, while no one has specifically explored how industrial coagglomeration affects manufacturing carbon emissions. Since the manufacturing sector covers a major part of both China’s carbon emissions and national economic output values, the low-carbon transformation of the manufacturing industry is crucial for China to accomplish both economic high-quality growth and “dual-carbon” goals. Therefore, exploring whether and how China’s current industrial coagglomeration level has a significant impact on manufacturing carbon emissions are of great importance.

Regarding the possible impact mechanism of industrial coagglomeration on manufacturing carbon emissions, first, according to the theory of agglomeration economy, industrial coagglomeration gathers various enterprises together, which can reduce information and transaction costs, facilitate enterprises‘ sharing of knowledge and technology, and strengthen their exchanges, cooperation, and competition [22]. Effective industrial coagglomeration is beneficial for the generation of economies of scale, knowledge spillover, cooperation, sharing, and benign competition effects [22]. Second, as an intermediate input sector, producer service industry runs through the entire manufacturing industry chain. Its coagglomeration with the manufacturing industry can help refine the market division of labor and optimize resource allocation, thus producing a specialization effect [23]. The above effects are conducive to improving the manufacturing carbon emission efficiency; that is, reducing the manufacturing carbon emissions of the unit production scale. However, the improvement of manufacturing carbon emission efficiency is not always beneficial for total carbon emission reduction. Driven by profits, many enterprises may enlarge their production scale, which may result in an energy rebound effect [24], thus increasing the total manufacturing carbon emissions. Meanwhile, coagglomeration itself is usually accompanied by a rise in the enterprise number and scale, which will also cause more manufacturing carbon emissions in the agglomeration regions [23], namely, the carbon emission increase effect brought by scale expansion. Third, due to resource constraints, when the enterprise number and scale exceed the carrying capacity of the agglomeration regions and reach a saturation state, a crowding effect will occur [22]. Due to the malignant resource competition, production costs will rise and the benign agglomeration effects of industrial coagglomeration in the regions will weaken, which is adverse to lowering manufacturing carbon emissions. Overall, the economies of scale, cooperation, sharing, benign competition effects, carbon emission increase effect brought by scale expansion, energy rebound effect, and crowding effect can coexist in the agglomeration regions, but whether industrial coagglomeration helps decrease manufacturing carbon emissions is determined by the dominant effects. Moreover, it is worth noting that industrial coagglomeration is a complicated dynamic evolution process. Because enterprise size, resource allocation effectiveness, technology spillover level, innovation degree, and firms’ collaboration and competition level differ at each stage of coagglomeration, the dominant carbon emission effects of industrial coagglomeration may be distinct at each stage [23]. Therefore, industrial coagglomeration is very likely to have a nonlinear effect on manufacturing carbon emissions, which varies with the coagglomeration level. However, such potential nonlinear influencing mechanisms of industrial coagglomeration on manufacturing carbon emissions from the viewpoint of coagglomeration’s dynamic evolution has been ignored by scholars.

Additionally, industrial coagglomeration may also indirectly impact manufacturing carbon emissions via green innovation. Some studies have shown that industrial coagglomeration might affect green innovation, which is a critical factor for undergoing manufacturing low-carbon transformation [25,26]. Moderate industrial coagglomeration can pool innovation resources, make it easier for upstream and downstream enterprises in an industrial chain to cooperate, and enhance technology spillover [22,23]. These factors are beneficial for the improvement of green innovation. Studies by Lin et al. [27] and Wu [28] support that industrial coagglomeration promotes green innovation. However, resource misallocation, enterprise chain extrusion, and low-efficiency balance result from low-level industrial coagglomeration, and the crowding effect caused by overly industrial coagglomeration may also hinder green innovation [23,29,30,31]. Zeng et al. [30] and Zhang et al. [23] hold that, at present, China’s industrial coagglomeration is in a low-efficiency stage, which is not conducive to enhancing green innovation. As for the connection between green innovation and manufacturing carbon emissions, on the one hand, green innovation can promote the improvement and application of clean energy, green technology, and energy-saving equipment, which helps to lower manufacturing carbon emissions from the source and production process. Studies by Lee and Min [32], Lu et al. [15], Mandal and Pal [33], and Zhu et al. [34] all support that green innovation aids in decreasing carbon emissions. On the other hand, some research also demonstrates that innovation is not always helpful for manufacturing carbon emission abatement [35]. First, although green innovation can enhance energy efficiency, it may lead to increased energy consumption by reducing the energy cost of production, which is referred to as the rebound effect [36]. The rebound effect will greatly diminish the emission reduction effect and even cause a rise in manufacturing carbon emissions [23]. Second, a portion of the funds utilized for conventional innovation will be squeezed by developing green technologies and purchasing green innovative equipment, which may indirectly weaken the emission abatement effect of traditional innovation. Hence, the improvement of green innovation is a double-edged sword to manufacturing carbon emissions, and it remains to be tested if China’s current green innovation level can lower manufacturing carbon emissions. As industrial coagglomeration may affect green innovation, and both have potential to impact manufacturing carbon emissions, it is very rational to speculate that there may exist an underlying indirect impact mechanism for industrial coagglomeration to affect manufacturing carbon emissions through green innovation. Few scholars have integrated these three factors for thorough research.

Overall, relevant studies have primarily concentrated on the effect of single industrial agglomeration on carbon emissions, or the relationships between two of the three factors of industrial coagglomeration, green innovation, and carbon emissions. No one has investigated the nonlinear link between industrial coagglomeration and manufacturing carbon emissions from the dynamic evolution perspective of coagglomeration, let alone incorporating industrial coagglomeration, green innovation, and manufacturing carbon emissions into one framework and further investigating the potential mediating mechanism by which green innovation affects the connection between industrial coagglomeration and manufacturing carbon emissions. Those research gaps provide us with space for exploration.

**Figure 1:**
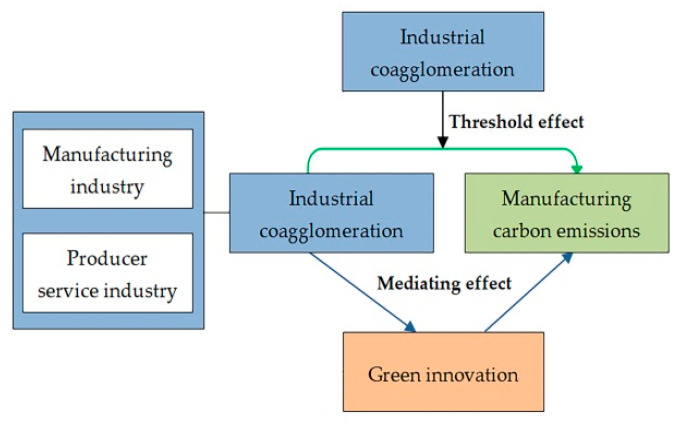
The research framework.

Given the above research deficiencies, from the dynamic evolution perspective of coagglomeration, this study explored whether industrial coagglomeration nonlinearly impacts manufacturing carbon emissions and further analyzed the direct and indirect (via green innovation) function mechanisms. Figure 1 shows the research framework. The conclusions can enrich the related theories of agglomeration economy and environmental ecology, fill the current research gaps, and provide empirical references for countries in terms of industrial layout, green innovation, and carbon emission abatement.

The research contributions mainly lie in three aspects. First, we designed an extended STIRPAT model to test the link between industrial coagglomeration and manufacturing carbon emissions, which enriches the theoretical system relevant to agglomeration economy and environmental ecology. Second, from the dynamic evolution perspective of coagglomeration, a new panel-threshold-STIRPAT model was developed to verify the nonlinear impact of industrial coagglomeration on manufacturing carbon emissions. Those investigations help us uncover the nonlinear influencing mechanism of industrial coagglomeration on manufacturing carbon emissions. Third, we first combined industrial coagglomeration, green innovation, and manufacturing carbon emissions into one system, and then built mediating-STIRPAT models to examine the different intermediary roles of green innovation in the relationship between industrial coagglomeration and manufacturing carbon emissions in the various evolutionary stages of coagglomeration. It was verified that industrial coagglomeration affects manufacturing carbon emissions both directly and indirectly (via green innovation).

The main work is as follows: Section 2 represents the related theoretical and econometric models. Section 3 provides the empirical results. Section 4 discusses the detailed causes of the results and future research. Section 5 reports conclusions and some policy implications.

## 2. Methods and Data

### 2.1. Extended STIRPAT Model

The IPAT model built by Ehrlich and Holdren in 1971 has been widely adopted by academics to explore man-made environmental influencing factors because of its simplicity and effectiveness [23,37]. The model indicates that the environmental impact (*I*) is determined by population (*P*), affluence (*A*), and technical level (*T*), respectively. The connection between the four variables is expressed by the following identical equation:

(1) I=P×A×T

Based on this, Waggoner and Ausubel extended the IPAT identical equation by decomposing the technology level into the product of technology consumed per unit of GDP (*C*) and the impact of each unit of technology on the environment (*T*) and established the identical equation, namely [38]:

(2) Im=P×A×C×T

This explores the leverage effect of a combination of influencing factors on environmental impact. Compared with the IPAT model, the ImPAC model can more clearly present the impact of consumption and production processes on the environment in the economic system. Nevertheless, it shares some common defects with the IPAT model. For example, when analyzing the problem by changing one influencing factor while keeping other factors fixed, the result obtained is the proportional effect of the changing influencing factor on dependent variables, which is inconsistent with the actual situation [39]. To overcome that drawback, Dietz and Rosa expressed the IPAT model as a random form and developed a STIRPAT model, which can examine the non-proportional effect of the impact factors on the environment; thus more effectively identifying the intricate connections between variables [40]. The model is expressed by

(3) $$I_{it}=\alpha \times P_{it}^{\theta }\times A_{it}^{\gamma }\times T_{it}^{\eta }\times \varepsilon_{it}$$

where θ, γ, and η are estimated parameters of P, A, and T. ε, t, i, α denote a random error term, time, region, and a constant term, respectively. Scholars have widely employed this model to test the driving factors of the environmental impact incorporating carbon emissions [41]. Referring to previous studies [39,40,41,42], we introduced the variable of industrial coagglomeration and developed a new model, which can be expressed as follows:

(4) $$I_{it}=\alpha \times P_{it}^{\theta }\times A_{it}^{\gamma }\times T_{it}^{\eta }\times {\mathrm{Coagg}}_{it}^{\beta }\times \varepsilon_{it}$$

where *Coagg* is the industrial coagglomeration level of manufacturing and producer service industries. β represents the elasticity of industrial coagglomeration on environmental impact. Meanwhile, to prevent the heteroscedasticity issue, we simultaneously took the logarithm of both sides of Equation (4) and developed the following panel data model:

(5) $$\ln I_{it}=\ln\alpha +\beta \ln{\mathrm{Coagg}}_{it}+\theta \ln P_{it}+\gamma \ln A_{it}+\eta \ln T_{it}+\delta \ln X_{it}+\ln\varepsilon_{it}$$

In this study, the manufacturing carbon emission level (*Mce*) was utilized to represent environmental impact (*I*). Population size (*Ps*), economic level (*El*), and green innovation (*Gi*) were adopted to reflect population (*P*), affluence (*A*), and technical level (*T*), respectively. Additionally, referencing prior research [9,41,43,44], we selected foreign direct investment (*Fdi*), energy intensity (*Ei*), and cleaning index of energy consumption structure (*Ecsci*) as control variables, expressed synthetically as *X*. Then, the model can be described as follows:

(6) $$\begin{array}{l} \ln{\mathrm{Mce}}_{it}=\ln\alpha +\beta \ln{\mathrm{Coagg}}_{it}+\theta \ln Ps_{it}+\gamma \ln El_{it}+\eta \ln Gi_{it}+\pi \ln Od_{it}+\phi \ln{\mathrm{Ecsci}}_{it}\\ +\eta \ln Ei_{it}+\ln\varepsilon_{it} \end{array}$$

### 2.2. Panel-Threshold-STIRPAT Model

The panel threshold model constructed by Hansen is a typical model investigating the nonlinear links between variables [45]. To explore if the influence of industrial coagglomeration on manufacturing carbon emissions is nonlinear, setting industrial coagglomeration as the threshold variable, the following single-threshold panel model was established:

(7) $$\begin{array}{l} \ln Mce_{it}=\ln\alpha +\beta_{1}\ln Coagg_{it}\cdot I(\ln Coagg_{it}\leq \gamma )+\beta_{2}\ln Coagg_{it}\cdot I(\ln Coagg_{it}>\gamma )\\ +\eta \ln Gi_{it}+\sum_{k=1}^{n}\theta_{k}\ln X_{it}+\ln\varepsilon_{it} \end{array}$$

where *Mce* stands for manufacturing carbon emission level, *Coagg* represents the coagglomeration degree of manufacturing and producer service industries, *Gi* denotes green innovation level, γ is a threshold value, *X* reflects the control variable, n is the number of control variables, t and i indicate time and region, and $\beta_{1}$, $\beta_{2}$, η, and $\theta_{k}$ represent the parameters to be estimated. The panel double- and triple-threshold models can be developed by extending Equation (7) as follows:

(8) $$\begin{array}{l} \ln Mce_{it}=\ln\alpha +\beta_{1}\ln Coagg_{it}\cdot I(\ln Coagg_{it}\leq \gamma_{1})+\beta_{2}\ln Coagg_{it}\cdot I(\gamma_{1}<\ln Coagg_{it}\leq \gamma_{2})\\ +\beta_{3}\ln Coagg_{it}\cdot I(\ln Coagg_{it}>\gamma_{2})+\eta \ln Gi_{it}+\sum_{k=1}^{n}\theta_{k}\ln X_{it}+\ln\varepsilon_{it} \end{array}$$

(9) $$\begin{array}{l} \ln Mce_{it}=\ln\alpha +\beta_{1}\ln Coagg_{it}\cdot I(\ln Coagg_{it}\leq \gamma_{1})+\beta_{2}\ln Coagg_{it}\cdot I(\gamma_{1}<\ln Coagg_{it}\leq \gamma_{2})+\beta_{3}\\ \ln Coagg_{it}\cdot I(\gamma_{2}<\ln Coagg_{it}\leq \gamma_{3})+\beta_{4}\ln Coagg_{it}\cdot I(\ln Coagg_{it}>\gamma_{3})+\eta \ln Gi_{it}+\sum_{k=1}^{n}\theta_{k}\ln X_{it}+\ln\varepsilon_{it} \end{array}$$

where $\gamma_{i}$ are threshold values and $\gamma_{1}<\gamma_{2}<\gamma_{3}$, and other symbols have the same meanings as those of Equation (7).

### 2.3. Mediation-STIRPAT Models

To further test if there is a mediating effect for green innovation between industrial coagglomeration and manufacturing carbon emissions, we built the following mediation models based on Wang et al. and Zhang et al.’s practices [23,46]:

Total effect model:

(10) $$\ln Mce_{it}=c\ln Coagg_{it}+\sum_{k=1}^{n}\theta_{k}\ln X_{it}+e_{1}$$

Indirect effect model:

(11) $$\ln Gi_{it}=a\ln Coagg_{it}+\sum_{k=1}^{n}\theta_{k}\ln X_{it}+e_{2}$$

Direct effect model:

(12) $$\ln Mce_{it}=c^{\prime }\ln Coagg_{it}+b\ln Gi_{it}+\sum_{k=1}^{n}\theta_{k}\ln X_{it}+e_{3}$$

First, the significance of the parameter c in Equation (10) needs to be assessed. If it is significant, we then move to step two, investigating the significance of a and b. If they are both significant, green innovation is an intermediary between industrial coagglomeration and manufacturing carbon emissions. If a or b is not significant, a Sobel test should be performed to determine if a mediation effect exists or not [23,46,47]. Next, the significance of parameter $c^{\prime }$ of Equation (12) also needs to be tested. If it is significant, industrial coagglomeration has both direct and indirect effects on manufacturing carbon emissions and green innovation is a partial intermediary. If not, there only exists an indirect effect via green innovation and green innovation is a complete intermediary.

### 2.4. Variables and Data

#### 2.4.1. Dependent Variable

Manufacturing carbon emission level (ln*Mce*) is the dependent variable. As manufacturing emissions in China are mainly CO_2_ emissions, this study used the CO_2_ emissions of the manufacturing industry to evaluate manufacturing carbon emissions. The data was from the database of China Emission Accounts and Datasets (CEADs), which adopted the sectoral approach of the Intergovernmental Panel on Climate Change (IPCC) to calculate the CO_2_ emissions of the manufacturing industry based on China’s provincial CO_2_ emission inventory.

#### 2.4.2. Explanatory Variables

Industrial coagglomeration degree (ln*Coagg*) is an explanatory variable as well as a threshold variable. Industrial coagglomeration measures the coagglomeration level of the regional producer service industry and manufacturing industry. Based on the practice of prior studies [23,30], the industrial coagglomeration level is calculated by the following Equation:

(13) $$\ln Coagg_{it}=\ln\left[\left(1-\frac{\left|Magg_{it}-Sagg_{it}\right|}{Magg_{it}+Sagg_{it}}\right)+\left(Magg_{it}+Sagg_{it}\right)\right]$$

where *Coagg* denotes the industrial coagglomeration level, *Magg* is the agglomeration degree of the manufacturing industry, *Sagg* is the agglomeration degree of the producer service industry, *t* indicates time, and *i* represents region. Both *Magg* and *Sagg* are calculated by location entropy based on the number of employed persons in urban units at year-end. The subindustries of the producer service industry are as follows: “leasing and business services industry”, “finance industry”, “scientific research and technical service industry”, “transportation, warehousing, and postal industry”, and “information transmission, software and information technology service industry” [48,49].

#### 2.4.3. Intermediary Variable

Green innovation level (ln*Gi*) is the intermediary variable. Patent number, including patent application count and patent authorization count, is the most direct reflection of innovation level [27]. As not all applied patents can be authorized, the number of patent authorizations can better reveal the regional innovation degree [23]. Hence, the number of green patent authorizations (by authorization year) was employed to assess the regional green innovation level. In 2010, the World Intellectual Property Organization issued the international patent classification green inventory. According to that green inventory, we acquired the number of green patent authorizations of each province by cleaning and filtering the patent data download from the China National Intellectual Property Administration (CNIPA).

#### 2.4.4. Control Variables

Referencing prior research, the following variables were chosen as control variables [9,41,43,44]. (1) Population size (*Ps*): Population growth usually causes a rise in domestic carbon emissions [34]; thus it is very likely that population size positively impacts manufacturing carbon emissions. The number of permanent residents at the end of the year in each province is utilized to assess the regional population size. (2) Economic level (*El*): Regions with greater economic levels usually have a better foundation for green innovation and environmental governance [23], so the economic level tends to negatively affect manufacturing carbon emissions. We adopted the real GDP per unit capital, measured by the ratio of the real GDP to gross fixed capital formation in constant price (2008 is the base period), to evaluate the economic level. (3) Open degree (*Od*): Foreign direct investment (*FDI*) is a reflection of the open degree. The pollution halo hypothesis holds that FDI can bring frontier technology to the host countries, which helps to enhance production efficiency; thus decreasing manufacturing carbon emissions. However, the pollution paradise hypothesis shows that FDI is a channel for advanced countries to transfer high-carbon and heavy-pollution industries to other countries, which intensifies the manufacturing carbon emissions of the host countries [22]. We chose the proportion of foreign capital to the paid-in capital of industrial enterprises to weigh the open degree. (4) Cleaning index of energy consumption structure (*Ecsci*): Since the carbon emission output per unit of dirty energy is significantly higher than that of clean energy, the cleaning index of energy consumption structure negatively impacts manufacturing carbon emissions [43] and its calculation formula is as follows: The cleaning index of energy consumption structure = 1 − (coal consumption/total energy consumption). (5) Energy intensity (*Ei*): The more energy used per unit output value means that more carbon emissions will be emitted if the output scale remains the same. The ratio of energy industry investment to GDP was utilized to evaluate energy intensity, and the energy intensity tends to positively affect manufacturing carbon emissions [41].

Data for 30 provinces of China between 2010 and 2019 were utilized. Hong Kong, Taiwan, Tibet, and Macao were not incorporated because of data collection limitations. The data were acquired from the National Bureau of Statistics, Carbon Emission Accounts for Emerging economies (CEADs), China Statistical Yearbook, and China Energy Statistics Yearbook. The variables’ descriptive statistical results are displayed in Table 1.

## 3. Results

### 3.1. Unit Root Test and Multicollinearity Check

To reduce the likelihood of pseudo regression, we examined the unit root of each variable by adopting the Levin–Lin–Chu (LLC) approach [50], whose original hypothesis is that there is a “unit root” for the variable. The findings in Table 2 suggest that at a 1% significance level, the original hypotheses for all variables are rejected. It means that no variable has a unit root. Additionally, since none of the variables’ variance inflation factor values are larger than 10, there is also no multicollinearity issue with the variables.

### 3.2. Industrial Coagglomeration’s Threshold Effect Regarding Its Impact on Manufacturing Carbon Emissions

#### 3.2.1. Threshold Effect Tests

**Figure 2:**
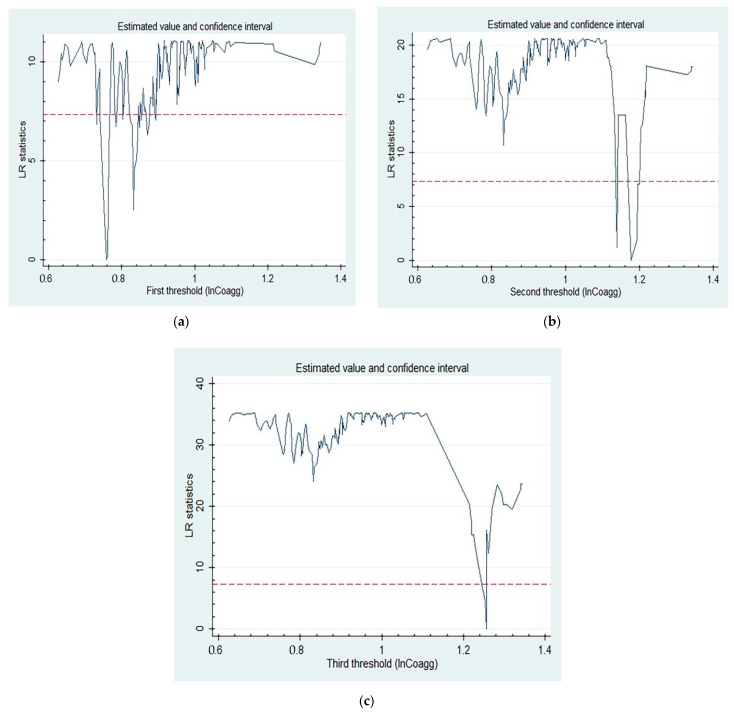
The LR graphs for the threshold assessment of ln*Coagg*: (**a**) the first threshold assessment, (**b**) the second threshold assessment, (**c**) the third threshold assessment.

To clarify whether there are thresholds for the influence of industrial coagglomeration on manufacturing carbon emissions, we perform threshold effect significance and authenticity tests. The results shown in Table 3 reflect that, when setting industrial coagglomeration (ln*Coagg*) as a threshold variable, the single-, double-, and triple-threshold effects are significant at a level of at least 5%. The likelihood ratio (LR) test in Figure 2 suggests that all thresholds pass the threshold effect authenticity test. Therefore, three thresholds exist and a panel triple-threshold regression model should be conducted.

#### 3.2.2. Results of Panel Threshold Regression

According to the panel triple-threshold regression results in Table 4, the influence of industrial coagglomeration on manufacturing carbon emissions varies with the coagglomeration’s dynamic evolution. There are three threshold values for ln*Coagg*, namely, 0.762, 1.176, and 1.256, respectively. As ln*Coagg* is no more than 0.762, industrial coagglomeration significantly and negatively impacts manufacturing carbon emissions and the regression coefficient is −0.980. When industrial coagglomeration hits the first threshold value of 0.762, the effect remains significantly negative, but the regression coefficient decreases to −0.728. Meanwhile, as ln*Coagg* further rises to the second threshold value of 1.176 and even the third threshold value of 1.256, the coefficients decrease further and the effects become insignificant. These results reveal that industrial coagglomeration helps lower manufacturing carbon emissions, but the effect has a diminishing trend in marginal utility. Only when the industrial coagglomeration is below a certain threshold (ln*Coagg* ≤ 1.176) can its manufacturing carbon emission abatement effect be significant.

As for the control variables, green innovation does not help decrease manufacturing carbon emissions. The causes are twofold: first, green innovation can enhance energy efficiency and raise carbon productivity, but rising energy efficiency also drives enterprises to expand their production scale to boost profits. Consequently, more energy will be consumed, which results in an energy rebound effect and raises the total manufacturing carbon emissions [24]. Second, part of the funds allocated for traditional innovation may be diverted to the research of green technologies and the acquisition of green innovative equipment, which cuts the emission reduction effect of traditional innovation and indirectly causes more manufacturing carbon emissions. The growth of population size helps cut manufacturing carbon emissions, which is different from Li’s finding [43]. This might be because, although rising populations result in higher household carbon emissions, they also enable cluster areas to gather more talents. Increased talents, particularly high-end innovative talents, considerably improve regional innovation potential, which aids in the abatement of manufacturing carbon emissions [23]. A rise in economic level helps to decrease manufacturing carbon emissions, confirming that regions with higher economic levels often have a better foundation for green innovation and environmental governance [23], thus promoting the manufacturing carbon emission reduction. The open degree has an insignificant negative impact on manufacturing carbon emissions, which means that, in China, the pollution halo and pollution paradise hypotheses might coexist and their impacts on the manufacturing carbon emissions offset each other [51]. The cleaning index of energy consumption structure significantly and negatively affects manufacturing carbon emissions. This is because the usage of clean energy can emit fewer carbon emissions than dirty energy [43]. Energy intensity has a positive yet insignificant influence. It may be because higher energy consumption per unit output value will cause higher carbon emissions if the scale remains the same, but the rise in carbon emissions may also stimulate enterprises to improve their energy efficiency via using intelligent devices or advanced technologies, expanding scale, and realizing economies of scale, etc., which in turn offsets some of the manufacturing carbon emission increase effect caused by the rise in energy intensity. These factors cause the effect of energy intensity to be insignificant.

#### 3.2.3. The Regional Distribution of the Industrial Coagglomeration Level

**Figure 3:**
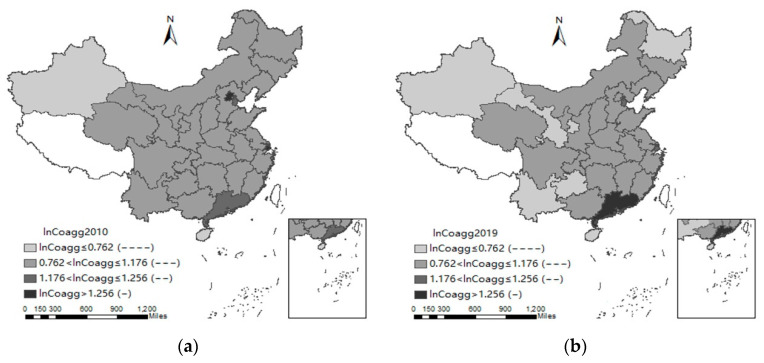
The regional distribution of industrial coagglomeration (ln*Coagg*) relative to the negative effect of industrial coagglomeration on manufacturing carbon emissions. “-”, “--”, “---”, and “----” represent the influence degree. The larger the number of “-”, the greater the effect. (**a**) 2010, (**b**) 2019.

To know whether or not the effect of industrial coagglomeration on manufacturing carbon emissions differs in various regions, this study further explored the regional distribution of industrial coagglomeration (ln*Coagg*). The results of model (1) reveal that only when ln*Coagg* is within the interval of [0, 1.176] can ln*Coagg* significantly lower manufacturing carbon emissions. Meanwhile, when ln*Coagg* is within the interval of [0, 0.762], the manufacturing carbon emission effect of ln*Coagg* is greatest. Figure 3 reflects, from 2010 to 2019, that the distribution of ln*Coagg* in China has marginally improved. The proportion of regions whose industrial coagglomeration level is significantly conducive to decreasing manufacturing carbon emissions increased from 83% to 90%, and the proportion of regions whose industrial coagglomeration level is within the interval having the greatest emission reduction effect rose from 10% to 20%. At present, the effect of industrial coagglomeration on manufacturing carbon emissions for 10% of provinces is not significant. For the remaining 90% of provinces, an increase in industrial coagglomeration level is conducive to decreasing manufacturing carbon emissions.

### 3.3. Green Innovation’s Mediating Effect between Industrial Coagglomeration and Manufacturing Carbon Emissions

The evaluation results for mediating effect models are displayed in Table 5. Model (2) investigated the total effect of industrial coagglomeration on manufacturing carbon emissions. The outcomes verify the significance of the parameter c and demonstrate that when ln*Coagg* is lower than 1.256, the total effect of industrial coagglomeration on manufacturing carbon emissions is significantly negative. Once ln*Coagg* exceeds 1.256, the effect becomes insignificant. Meanwhile, the impact has a diminishing trend in marginal utility. Then, we move on to step two, testing the significance of parameters a and b in Equations (11) and (12), thus Model (1) and Model (3) were conducted. According to the outcomes of the Hausman test, chi2(6) = 158.38 and *p* = 0.0000, Model (3) should adopt the fix-effect form. The panel fix-effect regression results for Model (3) indicate, at a 1% significance level, industrial coagglomeration negatively impacts green innovation, which is in line with Zeng’s research [30]. Meanwhile, the outcomes of Model (1) reflect that green innovation positively affects manufacturing carbon emissions at a 1% significance level. Overall, when ln*Coagg* is lower than 1.256, both parameters a and b are significant, thus green innovation is an intermediary between industrial coagglomeration and manufacturing carbon emissions. When ln*Coagg* is less than 1.176, as the parameter $c^{\prime }$ is significant, green innovation is a partial intermediary. When ln*Coagg* is between 1.176 and 1.256, as the parameter $c^{\prime }$ is not significant, green innovation is a complete mediator. Additionally, a Sobel test was also carried out in this test. The Sobel z statistic is 0.002 and significant at a 5% level, which further confirms green innovation’s mediating effect.

### 3.4. Robustness Tests

To guarantee the stability of the findings, we additionally ran some robustness tests. The robustness test methods commonly utilized by scholars include changing evaluation indicators of core variables, lagging key variables, and deleting control variables. First, referencing the research of Zhang et al. [23], we carried out the first robustness test by reassessing the intermediary variable (ln*Gi*) with the application count of green patents and re-estimated the regression results of models (1)–(3). The test outcomes are replicated in Table 6. Second, according to the practices of Zeng [30], Wang and Luo [51], and Zhang et al. [23], the second robustness test was performed by deleting control variables and lagging the explanatory variable ln*Coagg* by one year, which can effectively alleviate the possible endogenous problems between variables [44,52]. The re-estimated findings of models (1)–(3) are demonstrated in Table 7. The outcomes of robustness tests 1 and 2 reflect that the threshold effect of industrial coagglomeration and the mediating effect of green innovation still exist. Meanwhile, the significance and symbols of the coefficients for key variables, and the threshold number and values for ln*Coagg*, change slightly but not considerably. Hence, the conclusions of our study are stable.

## 4. Discussion

This research puts industrial coagglomeration, green innovation, and manufacturing carbon emissions into the same system and examines the connections among them from the dynamic evolution perspective of coagglomeration. First, we introduced industrial coagglomeration and green innovation into the STIRPAT framework and established an extended STIRPAT model. By combining that model with the panel threshold model, we then built a panel-threshold-STIRPAT model to investigate the nonlinear impacts of industrial coagglomeration on manufacturing carbon emissions in coagglomeration’s varied evolution stages. Second, utilizing the mediation-STIRPAT models, we verified the intermediary role of green innovation between industrial coagglomeration and manufacturing carbon emissions. The conclusions enrich the related theories of agglomeration economy and environmental ecology, fill the current research gaps, and provide empirical references for countries in terms of industrial distribution, green innovation, and carbon emission abatement. The causes for the results are further discussed for a deeper understanding of the direct and indirect (via green innovation) influencing mechanism of industrial coagglomeration on manufacturing carbon emissions.

### 4.1. The Causes of Industrial Coagglomeration’s Threshold Effect

The results of the panel-threshold-STIRPAT model suggest that industrial coagglomeration nonlinearly affects manufacturing carbon emissions and the impact has significant threshold effects in terms of industrial coagglomeration level. This is because the dominant effects alter as the coagglomeration degree increases. At the relatively low-level stage of industrial coagglomeration, the gathering of enterprises in the industrial chains and their resources enables enterprises to save their information search, transportation, and transaction costs, and promotes the generation of the economies of scale, cooperation, sharing, benign competition effects, etc. [22,23], which helps increase manufacturing carbon productivity, thus lowering the manufacturing carbon emissions. Meanwhile, the enterprises of producer services in the agglomeration area can assist the manufacturing enterprises to realize more specialized market division and efficient resource allocation [53], which are advantageous for increasing their production efficiency and cutting down the manufacturing carbon emissions per unit of resources. Hence, when ln*Coagg* is lower than 0.762, industrial coagglomeration can significantly decrease manufacturing carbon emissions. As industrial coagglomeration deepens, production efficiency gradually increases. Many enterprises might enlarge their production scale to obtain additional profits, which results in an energy rebound effect [24], increasing total manufacturing carbon emissions. Meanwhile, an increase in the regional enterprise number and scale will also produce more manufacturing carbon emissions [23]. The carbon emission increase impact resulting from the energy rebound effect and the growth in scale weakens the carbon emission abatement impact resulting from the enhancement of production efficiency. Consequently, as the industrial coagglomeration level increases, although industrial coagglomeration helps to lower manufacturing carbon emissions, the influencing degree decreases. Moreover, due to resource constraints, once the industrial coagglomeration exceeds a certain threshold, the enterprise number will reach saturation, and a crowding effect will occur in the agglomeration regions [23]. Production costs will rise as a result of malignant resource competition [22]. The economies of scale, cooperation, sharing, and benign competition effects will weaken and the resulting manufacturing carbon emission abatement effect will also decrease. When the gap between the manufacturing carbon emission abatement effect and the manufacturing carbon emission increase effect results from the energy rebound effect, the increase of scale and the crowding effect is not large, and the negative influence of industrial coagglomeration on manufacturing carbon emissions becomes insignificant. Hence, as industrial coagglomeration deepens, although it helps to lower manufacturing carbon emissions, the function effect has a decreasing trend in marginal utility. Meanwhile, when ln*Coagg* surpasses 1.176, the negative impact of industrial coagglomeration on manufacturing carbon emissions becomes insignificant.

### 4.2. The Causes of Green Innovation’s Mediating Effect

The findings of the mediation-STIRPAT models reveal the vital intermediary role of green innovation between industrial coagglomeration and manufacturing carbon emissions. First, enhancing green innovation is not aided by a rise in industrial coagglomeration. The causes are twofold. On the one hand, manufacturing green innovation is characterized by large-scale investment, a protracted return period, and significant uncertainty. To effectively stimulate enterprises’ enthusiasm for green innovation, it needs to be embedded with perfect intellectual property rights, a complete legal system, and other productive services [54]. However, China’s industrial coagglomeration is mainly government-guided with low coagglomeration efficiency [30]. Many enterprises move into the agglomeration regions for policy rent-seeking and have superficial cooperation with other enterprises. As a result, industrial coagglomeration’s externalities to green innovation through the knowledge spillover effect or enterprises’ deep cooperation cannot be successfully played. On the other hand, for enterprises just entering the agglomeration regions, their infrastructure, such as equipment and plants for green innovation, need to be repurchased or rebuilt, which raises their green innovation costs [23]. The high innovation costs further hinder the improvement of enterprises’ green innovation. Second, green innovation leads to increased manufacturing carbon emissions. This is primarily caused by the energy rebound effect and the crowding out of traditional innovation investment by that of green innovation, which reduces traditional innovation’s impact on manufacturing emission abatement. As such reasons have been explained in detail in Section 3.2.2, they will not be repeated here.

Overall, green innovation intensifies manufacturing carbon emissions. However, industrial coagglomeration is not beneficial for green innovation, thereby indirectly decreasing manufacturing carbon emissions. Hence, green innovation serves as a vital intermediary between industrial coagglomeration and manufacturing carbon emissions. Moreover, at the relatively low-level stage of industrial coagglomeration (ln*Coagg* ≤ 1.176), as the gap between the manufacturing carbon emission decrease effect brought about by the economies of scale, cooperation, sharing, and benign competition effects is significantly greater than the manufacturing carbon emission increase effect resulting from the energy rebound effect and the increase of scale, industrial coagglomeration has both significant direct and indirect (via green innovation) impacts on manufacturing carbon emissions. Therefore, green innovation is a partial mediator. At the relatively high-level stage of industrial coagglomeration (1.176 < lnCoagg ≤ 1.256), with the intensification of the energy’s rebound effect, the gaps between the above increase effect and decrease effect gradually weaken and become insignificant. In this stage, the negative impact of industrial coagglomeration on manufacturing carbon emissions plays its role only via its disadvantageous influence on green innovation; therefore, green innovation is a complete mediator.

### 4.3. Limitations and Future Research

Several shortcomings still exist due to the limited research time and available data. Further research can be improved in the following areas. First, owing to the different pollution degrees of manufacturing subindustries and their distinct relevance to the subindustries of producer services, there may be industry heterogeneity in the connection between industrial coagglomeration and manufacturing carbon emissions. Subsequent research can distinguish the coagglomeration of different subindustries of producer services and manufacturing, and further examine whether the coagglomeration of distinct subindustries has different impacts on manufacturing carbon emissions. Meanwhile, it can further explore the coagglomeration of which subindustries of producer services and manufacturing has the most significant effect on the carbon emissions of the manufacturing industry or its subindustries. Second, a more comprehensive indicator can be explored to better measure the industrial coagglomeration level. Third, considering the fluidity of manufacturing carbon emissions, future studies can take the spatial autocorrelation issue into account and explore whether the influence of industrial coagglomeration on manufacturing carbon emissions has a spatial spillover effect.

## 5. Conclusions and Policy Implications

Based on panel data from 30 provinces of China between 2010 and 2019, the relationships between industrial coagglomeration, green innovation, and manufacturing carbon emissions were examined. From coagglomeration’s dynamic evolution perspective, by using panel-threshold-STIRPAT and mediation-STIRPAT models, this study investigated if industrial coagglomeration has a nonlinear influence on manufacturing carbon emissions and further analyzed the direct and indirect (via green innovation) function mechanisms. The following are the detailed conclusions and policy recommendations:

### 5.1. Conclusions

First, from the dynamic evolution perspective of coagglomeration, industrial coagglomeration has a nonlinear impact on manufacturing carbon emissions. Three threshold values for ln*Coagg* exist, namely, 0.762, 1.176, and 1.256. Industrial coagglomeration negatively affects manufacturing carbon emissions, but the effect has a diminishing trend in marginal utility as the coagglomeration level increases. Only when the industrial coagglomeration is below a certain threshold (ln*Coagg* ≤ 1.176) can its manufacturing carbon emission abatement effect be significant. Once it exceeds that threshold, the emission reduction effect becomes insignificant. At present, for 90% of regions in China, an increase in industrial coagglomeration level is conducive to decreasing manufacturing carbon emissions.

Second, green innovation serves as a vital intermediary between industrial coagglomeration and manufacturing carbon emissions. Green innovation intensifies manufacturing carbon emissions. However, industrial coagglomeration is not beneficial for green innovation, thereby indirectly decreasing manufacturing carbon emissions. Moreover, when ln*Coagg* is less than 1.176, green innovation is a partial intermediary. When ln*Coagg* is between 1.176 and 1.256, green innovation is a complete intermediary.

Third, the growth of population size, economic level, and cleaning index of energy consumption structure is beneficial for lowering manufacturing carbon emissions, whereas the effects of open degree and energy intensity on manufacturing carbon emissions are insignificant.

### 5.2. Policy Implications

Several policy recommendations for lowering manufacturing carbon emissions are proposed.

First, policymakers need to adopt differentiated industrial coagglomeration strategies according to the evolution stages of coagglomeration in each region. For provinces such as Tianjin, Shanghai, and Guangdong, the industrial coagglomeration level can be appropriately controlled or reduced to prevent excessive industrial coagglomeration in the future. Meanwhile, partial high-carbon manufacturing industries can be eliminated to make the industrial structure green. For other regions, the government should attach more importance to moderately increasing the level and efficiency of industrial coagglomeration. For example, based on the development needs of manufacturing sectors, modern producer service chains can be developed around the manufacturing industry chains to improve the matching between the producer services and the manufacturing industry; thus enhancing the efficient cooperation and interaction between them and maximizing the carbon emission reduction effect of industrial coagglomeration.

Second, the innovation-driven strategy can be further promoted to support the development of regional green innovation. For one thing, government departments can work to build information service platforms more effectively, which helps lower communication barriers between related departments of producer services and the manufacturing industry and promote the spillover and sharing effects of knowledge and technologies. Meanwhile, it is also beneficial for the construction of a supply–demand relationship network and the deepening of the division of labor and industry collaboration, which contributes to the improvement of the innovative resources’ utilization efficiency and regional green innovation level. Additionally, the authorities should optimize the innovation infrastructure construction and related services in the existing industrial clusters, and create a favorable environment for enterprises to engage in green innovation. By offering incentives or subsidies, authorities can lessen the economic pressure that green innovation places on manufacturing enterprises, igniting their enthusiasm for the research and utilization of green technologies and effectively decreasing manufacturing carbon emissions.

Third, talent introduction should be vigorously encouraged so that the manufacturing carbon emission abatement effect brought by a rise in population size can be strengthened due to the increase in gathered high-end talents. The promotion of firms’ joint production is also encouraged to increase the economic level and its emission abatement effect. Moreover, enterprises are encouraged to use more energy with higher cleanliness to lower carbon emissions at the source.

## Figures and Tables

**Table 1:** Variables’ descriptive statistical results.

Variables	Observations	Mean	Maximum	Minimum	Standard Deviation
ln*Mce*	300	4.324	6.171	1.126	0.909
ln*Coagg*	300	0.952	1.387	0.584	0.167
ln*Gi*	300	7.417	10.364	3.044	1.382
ln*Ps*	300	8.201	9.352	6.333	0.736
ln*El*	300	0.525	2.433	−0.352	0.354
ln*Od*	300	−2.901	−0.965	−5.467	0.987
ln*Ecsci*	300	−0.586	−0.012	−1.286	0.268
ln*Ei*	300	−3.145	−1.135	−5.538	0.873

**Table 2:** The unit root and multicollinearity test results of variables.

Variables	LLC Test (Trend)	VIF
ln*Mce*	−9.0286 ***	-
ln*Coagg*	−5.1665 ***	2.49
ln*Gi*	−6.7596 ***	4.40
ln*Ps*	−4.9522 ***	3.20
ln*El*	−6.2835 ***	1.78
ln*Od*	−10.8109 ***	2.46
ln*Ecsci*	−8.8136 ***	2.01
ln*Ei*	−5.6441 ***	3.58
Mean VIF	-	2.85

Note: *** *p* < 0.01.

**Table 3:** Results of the threshold effect significance test.

Models	Threshold Estimates	F-Value	*p*-Value	1%	5%	10%	95% Confidence Interval
Single-threshold	1.256	35.286 ***	0.000	21.064	9.322	5.596	[1.139, 1.256]
Double-threshold	1.176 1.256	18.066 **	0.030	24.306	14.080	7.092	[1.139, 1.201] [1.245, 1.256]
Triple-threshold	0.762	10.844 **	0.040	19.276	10.271	6.376	[0.732, 0.893]

Notes: *** *p* < 0.01, ** *p* < 0.05; bootstrap = 500, min = 10, seed = 22,689.

**Table 4:** Regression on the threshold effect.

Variable	Model (1)	Variable	Model (1)
ln*Coagg* ≤ 0.762	−0.980 *** (−2.71)	ln*Od*	−0.003 (−0.09)
0.762 < ln*Coagg* ≤ 1.176	−0.728 ** (−2.23)	ln*Ecsci*	−0.480 *** (−4.37)
1.176 < ln*Coagg* ≤ 1.256	−0.362 (−1.14)	ln*Ei*	0.012 (0.32)
ln*Coagg* > 1.256	−0.094 (−0.31)	Constant	12.592 ** (2.55)
ln*Gi*	0.141 *** (4.66)	Obs	300
ln*Ps*	−1.083 * (−1.73)	R-sq	0.5457
ln*El*	−0.087 ** (−2.11)	F statistics	11.54 ***

Notes: the t values are in parentheses; *** *p* < 0.01, ** *p* < 0.05, * *p* < 0.1; bootstrap = 500, min = 10, seed = 22,689.

**Table 5:** Regression on the mediating effect.

Variable	Total Effect (DEPVAR = ln*Mce*) Model (2)	Variable	Direct Effect (DEPVAR = ln*Mce*) Model (1)	Variable	Indirect Effect (DEPVAR = ln*Gi*) Model (3)
ln*Coagg* ≤ 1.139	−0.814 *** (−2.62)	ln*Coagg* ≤ 0.762	−0.980 *** (−2.71)	ln*Coagg*	−3.055 *** (−5.45)
1.139 < ln*Coagg* ≤ 1.256	−0.542 * (−1.77)	0.762 < ln*Coagg* ≤ 1.176	−0.728 ** (−2.23)	-	-
ln*Coagg* > 1.256	−0.226 (−0.77)	1.176 < ln*Coagg* ≤ 1.256	−0.362 (−1.14)	-	-
-	-	ln*Coagg* > 1.256	−0.094 (−0.31)	-	-
ln*Gi*	-	ln*Gi*	0.141 *** (4.66)	-	-
Constant	−6.905 * (−1.78)	Constant	12.592 ** (2.55)	Constant	−105.509 *** (14.12)
Control variables	Yes	Control variables	Yes	Control variables	Yes
Obs	300	Obs	300	Obs	300
R-sq	0.6171	R-sq	0.5457	R-sq	0.8025
F statistics	8.36 ***	F statistics	11.54 ***	F statistics	178.81 ***

Note: the t values are in parentheses; *** *p* < 0.01, ** *p* < 0.05, * *p* < 0.1; bootstrap = 500, min = 10, seed = 22,689.

**Table 6:** Results of robustness test 1.

Variable	Total Effect (DEPVAR = ln*Mce*) Model (4)	Variable	Direct Effect (DEPVAR = ln*Mce*) Model (5)	Variable	Indirect Effect (DEPVAR = ln*Gi*) Model (6)
ln*Coagg* ≤ 1.139	−0.814 *** (−2.62)	ln*Coagg* ≤ 0.762	−1.085 *** (−3.02)	ln*Coagg*	−2.945 *** (−4.67)
1.139 < ln*Coagg* ≤ 1.256	−0.542 * (−1.77)	0.762 < ln*Coagg* ≤ 1.176	−0.829 ** (−2.57)	-	-
ln*Coagg* > 1.256	−0.226 (−0.77)	1.176 < ln*Coagg* ≤ 1.256	−0.446 (−1.42)	-	-
-	-	ln*Coagg* > 1.256	−0.178 (−0.59)	-	-
ln*Gi*	-	ln*Gi*	0.120 *** (4.44)		
Constant	−6.905 * (−1.78)	Constant	10.834 ** (2.26)	Constant	−107.878 *** (−12.83)
Control variables	Yes	Control variables	Yes	Control variables	Yes
Obs	300	Obs	300	Obs	300
R-sq	0.6171	R-sq	0.5133	R-sq	0.7940
F statistics	8.36 ***	F statistics	11.28 ***	F statistics	169.54 ***

Note: the t values are in parentheses; *** *p* < 0.01, ** *p* < 0.05, * *p* < 0.1; bootstrap = 500, min = 10, seed = 22,689.

**Table 7:** Results of robustness test 2.

Variable	Total Effect (DEPVAR = ln*Mce*) Model (7)	Variable	Direct Effect (DEPVAR = ln*Mce*) Model (8)	Variable	Indirect Effect (DEPVAR = ln*Gi*) Model (9)
ln*Coagg*_t−1_ ≤ 0.762	−1.253 *** (−3.42)	ln*Coagg*_t−1_ ≤ 0.762	−0.816 ** (−2.18)	ln*Coagg*_t−1_	−7.825 *** (−7.48)
0.762 < ln*Coagg*_t−1_ ≤ 1.237	−0.996 *** (−3.15)	0.762 < ln*Coagg* _t−1_ ≤ 1.237	−0.541 (−1.64)	-	-
ln*Coagg*_t−1_ > 1.237	−0.654 ** (−2.13)	ln*Coagg*_t−1_ > 1.237	−0.206 (−0.64)	-	-
ln*Gi*	-	ln*Gi*	0.061 *** (3.84)	-	-
Constant	5.267 *** (17.29)	Constant	4.383 *** (11.66)	Constant	14.905 *** (14.89)
Control variables	No	Control variables	No	Control variables	No
Obs	300	Obs	300	Obs	300
R-sq	0.0592	R-sq	0.0168	R-sq	0.1724
F statistics	13.10 ***	F statistics	14.01 ***	F statistics	56.02 ***

Note: the t values are in parentheses; *** *p* < 0.01, ** *p* < 0.05; bootstrap = 500, min = 10, seed = 22,689.

## Data Availability

Publicly available datasets were analyzed in this study. These data can be found here: National Bureau of Statistics. Available online: https://data.stats.gov.cn/ (accessed on 15 September 2021); Big data research platform for China’s economy and society. Available online: https://data.cnki.net/Yearbook/Navi?type=type&code=A (accessed on 15 June 2022); China Emission Accounts and Datasets (CEADs). Available online: https://www.ceads.net/data/ (accessed on 20 December 2021).

## References

[1] Chen P., Gao J., Ji Z., Liang H., Peng Y. (2022). Do Artificial Intelligence Applications Affect Carbon Emission Performance?—Evidence from Panel Data Analysis of Chinese Cities. Energies.

[2] Cheng S., Fan W., Meng F., Chen J., Cai B., Liu G., Liang S., Song M., Zhou Y., Yang Z. (2020). Toward Low-Carbon Development: Assessing Emissions-Reduction Pressure among Chinese Cities. J. Environ. Manag..

[3] Zeng N., Jiang K., Han P., Hausfather Z., Cao J., Kirk-Davidoff D., Ali S., Zhou S. (2022). The Chinese Carbon-Neutral Goal: Challenges and Prospects. Adv. Atmos. Sci..

[4] Liu J., Yang Q., Ou S., Liu J. (2022). Factor Decomposition and the Decoupling Effect of Carbon Emissions in China’s Manufacturing High-Emission Subsectors. Energy.

[5] An Y., Zhou D., Yu J., Shi X., Wang Q. (2021). Carbon Emission Reduction Characteristics for China’s Manufacturing Firms: Implications for Formulating Carbon Policies. J. Environ. Manag..

[6] Hang Y., Wang Q., Zhou D., Zhang L. (2019). Factors Influencing the Progress in Decoupling Economic Growth from Carbon Dioxide Emissions in China’s Manufacturing Industry. Resour. Conserv. Recycl..

[7] Lyu R., Zhang C., Li Z., Li Y. (2022). Manufacturers’ Integrated Strategies for Emission Reduction and Recycling: The Role of Government Regulations. Comput. Ind. Eng..

[8] Howard E., Newman C., Tarp F. (2016). Measuring Industry Coagglomeration and Identifying the Driving Forces. J. Econ. Geogr..

[9] Meng X.-N., Xu S.-C. (2022). Can Industrial Collaborative Agglomeration Reduce Carbon Intensity? Empirical Evidence Based on Chinese Provincial Panel Data. Environ. Sci. Pollut. Res..

[10] Ellison G., Glaeser E.L. (1997). Geographic Concentration in U.S. Manufacturing Industries: A Dartboard Approach. J. Polit. Econ..

[11] Ellison G., Glaeser E.L., Kerr W.R. (2010). What Causes Industry Agglomeration? Evidence from Coagglomeration Patterns. Am. Econo. Rev..

[12] Venables A.J. (1996). Equilibrium Locations of Vertically Linked Industries. Int. Econ. Rev..

[13] Ke S., He M., Yuan C. (2014). Synergy and Co-Agglomeration of Producer Services and Manufacturing: A Panel Data Analysis of Chinese Cities. Reg. Stud..

[14] Lanaspa L., Sanz-Gracia F., Vera-Cabello M. (2016). The (Strong) Interdependence between Intermediate Producer Services’ Attributes and Manufacturing Location. Econ. Model..

[15] Lu N., Feng S., Liu Z., Wang W., Lu H., Wang M. (2020). The Determinants of Carbon Emissions in the Chinese Construction Industry: A Spatial Analysis. Sustainability.

[16] Xu W., Yang P., Xiao Y. (2022). Will the Special Economic Zone Contribute to Carbon Neutrality? Quasi-Natural Experimental Evidence from China. Appl. Econ. Lett..

[17] Huang Q., Hu Y., Luo L. (2022). Spatial Analysis of Carbon Dioxide Emissions from Producer Services: An Empirical Analysis Based on Panel Data from China. Environ. Sci. Pollut. Res..

[18] Chen D., Chen S., Jin H. (2018). Industrial Agglomeration and CO_2_ Emissions: Evidence from 187 Chinese Prefecture-Level Cities over 2005–2013. J. Clean. Prod..

[19] Peng H., Wang Y., Hu Y., Shen H. (2020). Agglomeration Production, Industry Association and Carbon Emission Performance: Based on Spatial Analysis. Sustainability.

[20] Sun Z., Liu Y. (2021). Does Industrial Agglomeration Promote Carbon Efficiency? A Spatial Econometric Analysis and Fractional-Order Grey Forecasting. J. Math..

[21] Li T., Han D., Feng S., Liang L. (2019). Can Industrial Co-Agglomeration between Producer Services and Manufacturing Reduce Carbon Intensity in China?. Sustainability.

[22] Zhang L., Mu R., Hu S., Zhang Q., Wang S. (2021). Impacts of Manufacturing Specialized and Diversified Agglomeration on the Eco-Innovation Efficiency—A Nonlinear Test from Dynamic Perspective. Sustainability.

[23] Zhang L., Mu R., Hu S., Yu J., Zhang J. (2022). Industrial Coagglomeration, Technological Innovation, and Environmental Pollution in China: Life-Cycle Perspective of Coagglomeration. J. Clean. Prod..

[24] Böhringer C., Rivers N. (2021). The Energy Efficiency Rebound Effect in General Equilibrium. J. Environ. Manag..

[25] Yan Y., Li J., Xu Y. (2021). Research on Industry Difference and Convergence of Green Innovation Efficiency of Manufacturing Industry in China Based on Super-SBM and Convergence Models. Math. Probl. Eng..

[26] Shi R., Cui Y., Zhao M. (2021). Role of Low-Carbon Technology Innovation in Environmental Performance of Manufacturing: Evidence from OECD Countries. Environ. Sci. Pollut. Res..

[27] Lin S., Chen Z., He Z. (2021). Intra-City Industrial Collaborative Agglomeration, Inter-City Network Connectivity and Green Technology Innovation. Sustainability.

[28] Wu H., Al-Turjman F., Rasheed J. (2022). Threshold Effect of Collaborative Agglomeration of Internet and High-Tech Industry on Green Innovation. Forthcoming Networks and Sustainability in the IoT Era.

[29] Yang H., Xu X., Zhang F. (2022). Industrial Co-Agglomeration, Green Technological Innovation, and Total Factor Energy Efficiency. Environ. Sci. Pollut. Res..

[30] Zeng W., Li L., Huang Y. (2021). Industrial Collaborative Agglomeration, Marketization, and Green Innovation: Evidence from China’s Provincial Panel Data. J. Clean. Prod..

[31] Yang N., Yuan X., Qin F., Qian F. (2022). Coagglomeration of Manufacturing and Producer Services: How Does It Affect Regional Innovation in China?. Appl. Spatial Anal..

[32] Lee K.-H., Min B. (2015). Green R&D for Eco-Innovation and Its Impact on Carbon Emissions and Firm Performance. J. Clean. Prod..

[33] Mandal A., Pal B. (2021). Effects of Green Innovation and Advertisement in an Imperfect Production-based Competitive Supply Chain under Two-tier Credit Facility. Math. Method Appl. Sci..

[34] Zhu J., Dou Z., Yan X., Yu L., Lu Y. (2022). Exploring the Influencing Factors of Carbon Neutralization in Chinese Manufacturing Enterprises. Environ. Sci. Pollut. Res..

[35] Danish, Ulucak R. (2021). Renewable Energy, Technological Innovation and the Environment: A Novel Dynamic Auto-Regressive Distributive Lag Simulation. Renew. Sust. Energ. Rev..

[36] Vélez-Henao J.-A., Font Vivanco D., Hernández-Riveros J.-A. (2019). Technological Change and the Rebound Effect in the STIRPAT Model: A Critical View. Energy Policy.

[37] Ehrlich P.R., Holdren J.P. (1971). Impact of Population Growth: Complacency Concerning This Component of Man’s Predicament Is Unjustified and Counterproductive. Science.

[38] Waggoner P.E., Ausubel J.H. (2002). A framework for sustainability science: A renovated IPAT identity. Proc. Natl. Acad. Sci. USA.

[39] Li Z., Li Y.B., Shao S.S. (2019). Analysis of Influencing Factors and Trend Forecast of Carbon Emission from Energy Consumption in China Based on Expanded STIRPAT Model. Energies.

[40] Dietz T., Rosa E.A. (1997). Effects of Population and Affluence on CO _2_ Emissions. Proc. Natl. Acad. Sci. USA.

[41] Huo T., Cao R., Du H., Zhang J., Cai W., Liu B. (2021). Nonlinear Influence of Urbanization on China’s Urban Residential Building Carbon Emissions: New Evidence from Panel Threshold Model. Sci. Total Environ..

[42] Chang K.L., Du Z.F., Chen G.J., Zhang Y.X., Sui L.L. (2021). Panel estimation for the impact factors on carbon dioxide emissions: A new regional classification perspective in China. J. Clean. Prod..

[43] Li W., Wang W., Wang Y., Qin Y. (2017). Industrial Structure, Technological Progress and CO_2_ Emissions in China: Analysis Based on the STIRPAT Framework. Nat. Hazards.

[44] Wang Z., Zhu Y. (2020). Do Energy Technology Innovations Contribute to CO_2_ Emissions Abatement? A Spatial Perspective. Sci. Total Environ..

[45] Hansen B.E. (1999). Threshold Effects in Non-Dynamic Panels: Estimation, Testing, and Inference. J. Econom..

[46] Wang W., Li Y., Lu N., Wang D., Jiang H., Zhang C. (2020). Does Increasing Carbon Emissions Lead to Accelerated Eco-Innovation? Empirical Evidence from China. J. Clean. Prod..

[47] MacKinnon D.P., Lockwood C.M., Hoffman J.M., West S.G., Sheets V. (2002). A Comparison of Methods to Test Mediation and Other Intervening Variable Effects. Psychol. Methods.

[48] Xie R., Yao S., Han F., Fang J. (2019). Land Finance, Producer Services Agglomeration, and Green Total Factor Productivity. Int. Reg. Sci. Rev..

[49] Waiengnier M., Van Hamme G., Hendrikse R., Bassens D. (2020). Metropolitan Geographies of Advanced Producer Services: Centrality and Concentration in Brussels. Tijds. Econ. Soc. Geog..

[50] Levin A., Lin C.-F., Chu C.-S.J. (2002). Unit Root Tests in Panel Data: Asymptotic and Finite Sample Properties. J. Econ..

[51] Wang X., Luo Y. (2020). Has Technological Innovation Capability Addressed Environmental Pollution from the Dual Perspective of FDI Quantity and Quality? Evidence from China. J. Clean. Prod..

[52] Li K., Fang L., He L. (2018). How Urbanization Affects China’s Energy Efficiency: A Spatial Econometric Analysis. J. Clean. Prod..

[53] Zhang T., Su P., Deng H. (2021). Does the Agglomeration of Producer Services and the Market Entry of Enterprises Promote Carbon Reduction? An Empirical Analysis of the Yangtze River Economic Belt. Sustainability.

[54] Shearmur R., Doloreux D. (2013). Innovation and Knowledge-Intensive Business Service: The Contribution of Knowledge-Intensive Business Service to Innovation in Manufacturing Establishments. Econ. Innov. New Technol..

